# The role of Nrf2 in acute and chronic muscle injury

**DOI:** 10.1186/s13395-020-00255-0

**Published:** 2020-12-08

**Authors:** Iwona Bronisz-Budzyńska, Magdalena Kozakowska, Paulina Podkalicka, Neli Kachamakova-Trojanowska, Agnieszka Łoboda, Józef Dulak

**Affiliations:** 1 Department of Medical Biotechnology, Faculty of Biochemistry, Biophysics and Biotechnology, Jagiellonian University, Gronostajowa 7, 30-387 Kraków, Poland; 2 Malopolska Centre of Biotechnology, Jagiellonian University, Kraków, Poland

**Keywords:** Nrf2, Duchenne muscular dystrophy, Skeletal muscle, Satellite cells, *mdx*, Inflammation, Regeneration, Cardiotoxin-induced injury

## Abstract

The nuclear factor erythroid 2-related factor 2 (Nrf2) is considered as a master cytoprotective factor regulating the expression of genes encoding anti-oxidant, anti-inflammatory, and detoxifying proteins. The role of Nrf2 in the pathophysiology of skeletal muscles has been evaluated in different experimental models, however, due to inconsistent data, we aimed to investigate how Nrf2 transcriptional deficiency (Nrf2^tKO^) affects muscle functions both in an acute and chronic injury. The acute muscle damage was induced in mice of two genotypes—WT and Nrf2^tKO^ mice by cardiotoxin (CTX) injection. To investigate the role of Nrf2 in chronic muscle pathology, *mdx* mice that share genetic, biochemical, and histopathological features with Duchenne muscular dystrophy (DMD) were crossed with mice lacking transcriptionally active Nrf2 and double knockouts (*mdx*/Nrf2^tKO^) were generated. To worsen the dystrophic phenotype, the analysis of disease pathology was also performed in aggravated conditions, by applying a long-term treadmill test. We have observed slightly increased muscle damage in Nrf2^tKO^ mice after CTX injection. Nevertheless, transcriptional ablation of Nrf2 in *mdx* mice did not significantly aggravate the most deleterious, pathological hallmarks of DMD related to degeneration, inflammation, fibrotic scar formation, angiogenesis, and the number and proliferation of satellite cells in non-exercised conditions. On the other hand, upon chronic exercises, the degeneration and inflammatory infiltration of the gastrocnemius muscle, but not the diaphragm, turned to be increased in Nrf2^tKO^*mdx* in comparison to *mdx* mice. In conclusion, the lack of transcriptionally active Nrf2 influences moderately muscle pathology in acute CTX-induced muscle injury and chronic DMD mouse model, without affecting muscle functionality. Hence, in general, we demonstrated that the deficiency of Nrf2 transcriptional activity has no profound impact on muscle pathology in various models of muscle injury.

## Background

Duchenne muscular dystrophy (DMD) is the most common form of muscular dystrophies [1], which affects one in 5000-6000 male births [2]. DMD is the lethal X-chromosome linked recessive genetic neuromuscular disorder, caused by mutations in the gene encoding dystrophin [1]. Dystrophin deficiency leads to progressive muscle weakness, severe muscular atrophy, cardiomyopathy, and respiratory impairments, the two latter being the leading causes of mortality among patients with DMD [2]. Dystrophin, a cytoskeletal protein, is a major structural element of the dystrophin-glycoprotein complex (DGC), which is responsible for maintaining cellular integrity by linking the sarcolemmal and actin cytoskeleton to the extracellular matrix component laminin [3]. The loss of dystrophin disrupts the complex resulting in sarcolemmal instability that makes cells more susceptible to damage and leads to necrosis of muscle fibers [3]. Consequently, it results in the activation of the innate immune system, excessive inflammatory response, and increased oxidative stress [4].

In the early stage of the inflammatory response, muscles are infiltrated by neutrophils and pro-inflammatory, phagocytic M1-like macrophages which are a rich source of Th1 cytokines, that promote the activation and chemotaxis of myeloid cells to damaged tissue. Moreover, cytokines affect proliferation, migration, and differentiation of muscle satellite cells (SCs), progenitors of mature skeletal muscle. Subsequently, the recruitment of anti-inflammatory and pro-regenerative subpopulation of M2-like macrophages is observed [5]. In addition to macrophages and neutrophils, other inflammatory cells, including T-lymphocytes (cytotoxic, helper) may contribute to disease progression [6].

Subsequently, the injury leads to muscle regeneration, a process that depends on activation and proliferation of SCs and their differentiation into myotubes and later on regenerating myofibres that are centrally nucleated and exhibit expression of embryonic myosin heavy chain (eMyHC) isoform. Eventually, in chronic injury, the continuous cycles of myofiber degeneration and regeneration induce exhaustion of SCs and substitution of muscle with fibroadipose tissue [4]. Additionally, oxidative stress with elevated production of reactive oxygen species (ROS) has been proposed as important contributors in the pathogenesis of the DMD in humans [7] and *mdx* mice (murine model of DMD) [8].

Recently, we have shown that expression of heme oxygenase-1 (HO-1, encoded by *Hmox1*), anti-inflammatory, and cytoprotective enzyme, is strongly elevated in muscles of *mdx* mice and muscle biopsies of DMD patients [9]. Genetic loss of HO-1 not only exacerbates dystrophic phenotype and inflammation in *mdx* mice [9] but also aggravates skeletal muscle injury in acute muscle damage model, i.e., following cardiotoxin (CTX) injection [10]. Expression of *Hmox1* is regulated, among others, by the redox-sensitive nuclear factor erythroid 2-related factor 2 (Nrf2, encoded by *Nfe2l2* gene) belonging to Cap “n” collar (Cnc)-bZIP (basic leucine zipper) family of transcription factors [11]. Nrf2 plays a cytoprotective role as a master regulator of genes encoding oxidative stress response and phase II detoxifying proteins by interacting with the anti-oxidant response element (ARE) sequence. Under normal circumstances, Nrf2 is sequestered in the cytoplasm by Kelch-like ECH-associating protein 1 (Keap1) through the Nrf2-Keap1 complex, which suppresses Nrf2 activity by targeting it for ubiquitination and degradation. In stressful conditions, Nrf2 dissociates from Keap1, translocates into the nucleus, and induces the expression of target genes [12]. The role of Nrf2 in skeletal muscle aging and adaptations to exercise through the regulation of mitochondrial function, maintaining the cellular redox balance, control of oxidative stress, influencing apoptotic signaling, and providing proper contractile properties has been demonstrated [13, 14]. Moreover, although the involvement of Nrf2 in DMD progression has been suggested [15–18], the possible protective mechanisms were not fully discovered. Therefore, we aimed to evaluate the impact of Nrf2 transcriptional deficiency on the acute muscle damage caused by CTX injection and chronic injury using a murine model of DMD—*mdx* mice.

## Methods

### Animal models

All animal procedures and experiments were performed in accordance with national and European legislation, after approval by the 1st and 2nd Institutional Animal Care and Use Committee (IACUC) in Kraków, Poland (approval numbers: 66/2013, 199/2018, 148/2020). Mice were kept in specific-pathogen-free (SPF) conditions with water and food available ad libitum under controlled temperature and humidity and 14 h/10 h (light/dark) cycles.

*Mdx* mice (C57BL/10ScSn-*Dmd*^*mdx*^/J) and control mice (C57BL/10ScSnJ, WT) were purchased from the Jackson Laboratory. The mice with disrupted *Nfe2l2* gene on C57Bl/6 J background, originally developed by Prof. Yamamoto [19] were provided by Prof. Antonio Cuadrado [20] and further demonstrated by us to be a transcriptional knockout (Nrf2^tKO^) [21]. To generate Nrf2^tKO^*mdx* mice (deficient for both dystrophin and transcriptionally active Nrf2), homozygous Nrf2^tKO^ male mice were crossed with homozygous *Dmd*^*mdx/mdx*^ female mice to obtain Nrf2^+/*−*^ Dmd^*mdx*/+^ female mice or Nrf2^+/*−*^ Dmd^*mdx*/Y^ male mice, which were subsequently bred together to generate Nrf2^tKO^*mdx* mice at mixed background C57BL/10ScSn and C57BL/6 J.

In the experiments, if not stated otherwise, 10-12-week-old male littermates or age-matched mice from generation F2 to F5 were used. Accordingly, the double knockout animals lacking both dystrophin and Nrf2 expression (Nrf2^tKO^Dmd^mdx/Y^) were compared to their *mdx* littermates (Nrf2^+/+^Dmd^mdx/Y^). Additionally, *mdx* mice were analyzed vs. WT (Nrf2^+/+^Dmd^+/Y^) mice and the comparison of Nrf2^tKO^ (Nrf2^tKO^Dmd^+/Y^) mice vs. WT mice was studied as well.

The generation of double knockouts was hence done accordingly to other studies in which *mdx* mice were crossed with relevant knockouts [9, 22–25]. Genotyping of animals was performed by PCR on the DNA isolated from the tails.

### CTX-induced injury

Male and female C57BL/6 J (WT and Nrf2^tKO^) mice at 8-15 weeks of age were used for the myoinjury experiment. Hind limbs of mice were shaved and gastrocnemius muscles (GM) were injected with 25 μl of 20 μmol/l cardiotoxin from Mozambique spitting cobra (*Naja mossambica mossambica*; Sigma-Aldrich), while control mice were injected with saline. Animals were provided with analgesia (50 μl, 0.03 mg/ml buprenorphine) after injection and in the next 2 days. Mice were euthanized on the 1st, 3rd, 7th, 14th, and 28th day after CTX injury. Subsequently, plasma was taken and GM were harvested for further analyses.

### Treadmill test

To assess muscle functionality and performance, the treadmill test was performed as described previously [9, 24]. Additionally, to worsen the dystrophic phenotype, mice were subjected to chronic treadmill exercises. The training protocol started at the age of 4 weeks. After 3 days of acclimatization, mice underwent a run on a horizontal treadmill at 12 m/min for 30 min twice a week for 4 weeks, according to published protocols [26, 27]. Experiments were performed using the Exer-3/6 treadmill (Columbus Instruments).

### Forelimb grip strength test

Forelimb grip strength was assessed using a grip strength meter (GSM) with a triangular pull bar (Ugo Basile) according to the published protocol with modifications [28]. Briefly, the animals were gently held by the tail allowing them to grasp the grid using forelimbs. Afterward, mice were moved horizontally toward the bar and pulled back until the grip was released. The measurements were repeated 5 times with a 1-min break in between. The results were calculated as an average from 5 measurements, normalized to body weight, and expressed as N/kg BW.

### Plasma creatine kinase and lactate dehydrogenase measurement

Plasma was obtained by blood collection from *vena cava* to heparin-coated tubes followed by centrifugation at 1000×*g* for 10 min at 4 °C just before the terminal procedure and collection of GM. The activity of CK and LDH were measured using diagnostic Liquick Cor-CK and Liquick Cor-LDH kit, respectively (P.Z. CORMAY) following the manufacturer’s instruction using plasma diluted 10 times for measurements.

### Histological analysis

GM were dissected, immediately fixed in 10% formalin, dehydrated, embedded in paraffin, and cut into 4 μm sections. Subsequently, sections were deparaffinized, rehydrated, and subjected to histological stainings. Hematoxylin and eosin staining (H&E, Sigma-Aldrich) was performed to visualize inflammation and regenerating myofibers according to standard protocols. For Masson’s trichrome staining assessing collagen content (fibrosis evaluation), sections were fixed overnight in Bouin’s solution and sequentially treated with Biebrich scarlet-acid fuchsin, phosphotungstic acid/phosphomolybdic acid, and aniline blue (Sigma-Aldrich, according to the vendor’s instructions). Inflammation, muscle damage, fibrosis, and regeneration scoring was done by a blinded experimenter using the following description of arbitrary scale:

- 0—no signs of inflammation/collagen deposition/regenerating fibers
- 1—any sign of inflammation, myofibre swelling/collagen deposition/regenerating fibers
- 2—signs of inflammation, myofibre swelling, and rhabdomyolysis/collagen deposition/regenerating fibers are well visible and significant but occupy less than half of the field of view
- 3—signs of inflammation, myofibre swelling, and rhabdomyolysis/collagen deposition/regenerating fibers take up more than half of the field of view
- 4—signs of inflammation, myofibre swelling, and rhabdomyolysis/collagen deposition/regenerating fibers take up all field of view

### Immunohistofluorescent stainings

GM were harvested and snap-frozen in optimal cutting temperature compound (OCT, Leica) in liquid nitrogen-chilled isopentane and stored at −80 °C until processed. Frozen tissues were cryosectioned (10 μm) using a cryostat (Leica) and placed on glass slides coated previously with poly-l-lysine (Sigma-Aldrich).

For evaluation of necrotic fibers (accumulating IgG and IgM) and regenerating fibers (positive for embryonic myosin chain, eMyHC), sections were blocked with 10% goat serum (Sigma-Aldrich), 5% bovine serum albumin (BSA, BioShop), and mouse-on-mouse (M.O.M.^TM^, Vector Laboratories) for 1 h at room temperature, and incubated with rat anti-mouse laminin α2 (1:500; 4H8-2, Abcam) and mouse anti-mouse eMyHC (1:100, F1.652, DSHB) primary antibodies for 1 h at 37 °C. After three washes with PBS (5 min each), the sections were incubated with goat anti-rat Alexa Fluor 568 (1:1000, A-11077, Thermo Fisher Scientific) and goat anti-mouse IgG/IgM/IgA Alexa Fluor 488 (1:50, A-10667, Thermo Fisher Scientific) secondary antibodies for 1 h at 37 °C. Paired-box 7 (Pax7) level was checked on frozen cryosections fixed by 4% paraformaldehyde (Santa Cruz Biotechnology) and cold methanol (Avantor Performance Materials Poland S.A.). After antigen retrieval, samples were blocked for 30 min with 2.5% BSA and for the next 30 min with M.O.M.^TM^. Following two washes with PBS, sections were stained overnight at 4 °C with mouse anti-mouse Pax7 (1:100, Pax7, DSHB) and rabbit anti-mouse laminin α2 (1:1000, L9393, Sigma-Aldrich) primary antibodies diluted in 0.1% BSA. Secondary stains were done using goat anti-mouse Alexa Fluor 488 (1:500, A11008, Thermo Fisher Scientific) and goat anti-rabbit Alexa Fluor 568 (1:500, A-11077, Thermo Fisher Scientific) antibodies diluted in 0.1% BSA. Finally, sections were washed with PBS 3×5 min, during the last washing step nuclei were stained with Hoechst 33258 (10 μg/ml, Sigma-Aldrich) followed by mounting the slides with fluorescence mounting medium (Dako). Images were acquired using a fluorescent microscope (Leica DMI6000B) and analyzed in the *ImageJ* software. The number of necrotic or eMyHC-positive fibers was counted in 8 fields of view or within all injured sites of GM in the experiments with CTX injection, respectively. The percentage of necrotic/eMyHC^+^ myofibers was calculated in relation to the total number of myofibers. The ratio of Pax7^+^ nuclei/myofiber was estimated by counting Pax7^+^ nuclei and myofibers in at least 10 fields of view.

Gastrocnemius muscle cross-sectional area (CSA) and the mean fiber area were determined by semi-automatic muscle analysis using segmentation of histology (SMASH) [29] based on immunofluorescent staining of laminin described above. Only the injured area of the muscle was examined by the experimenter blinded to the mouse genotype.

### Gene expression analysis by quantitative real-time PCR

Harvested skeletal muscle tissues were stored in RNA*later* RNA Stabilization Solution (Invitrogen) at −80 °C until processed. GM from 12-week-old mice were used to isolate RNA by homogenization in 1 ml of Qiazol Total RNA Isolation Reagent (Qiagen) using TissueLyser (Qiagen), following the manufacturer’s instructions. The concentration and quality of RNA were determined spectrophotometrically (NanoDrop, Thermo Fisher Scientific). To synthesize cDNA, the reverse transcription reaction was performed on 1 μg RNA using RevertAid reverse transcriptase (Thermo Fisher Scientific) or MystiCq® microRNA cDNA Synthesis Mix (Sigma-Aldrich). qPCR was performed with Applied Biosystems^TM^ StepOnePlus Real-Time PCR (Thermo Fisher Scientific) in a mixture containing cDNA, SYBR Green PCR Master Mix (SYBR Green qPCR Kit, Sigma-Aldrich), forward and reverse primers recognizing murine genes (Table 1), and muscle-specific murine microRNAs (Table 2). A universal reverse primer for miRNAs qPCR was supplied by a vendor. The relative quantification of gene expression was quantified based on the comparative Ct (threshold cycle value) method. Gene expression levels were calculated by normalizing to the level of housekeeping gene elongation factor 2 (*Eef2*) or constitutive small nuclear RNA U6 in the case of microRNA.

**Table 1:** Sequences of primers used for qPCR analysis

Gene name	Forward primer	Reverse primer
*Col1a1*	5′-CGATCCAGTACTCTCCGCTCTTCC-3′	5′-ACTACCGGGCCGATGATGCTAACG-3′
*Eef2*	5′-AGAACATATTATTGCTGGCG-3′	5′-CAACAGGGTCAGATTTCTTG-3′
*Il1b*	5′-CTGGTGTGTGACGTTCCCATTA-3′	5′-CCGACAGCACGAGGCTTT-3′
*Il6*	5′-AAAGAGTTGTGCAATGGCAATTCT-3′	5′-AAGTGCATCATCGTTGTTCATACA-3′
*Kdr*	5′-CGGCCAAGTGATTGAGGCAG-3′	5′-ATGAGGGCTCGATGCTCGCT-3′
*Myh3*	5′-TCTAGCCGGATGGTGGTCC-3′	5′-GATTGTAGGAGCCACGAAA-3′
*Myod1*	5′-GCTGCCTTCTACGCACCTG-3′	5′-GCCGCTGTAATCCATCATGC-3′
*Myog*	5′-CAGTACATTGAGCGCCTACAG-3′	5′-GGACCGAACTCCAGTGCAT-3′
*Tgfb1*	5′-GGATACCAACTATTGCTTGAG-3′	5′-TGTCCAGGCTCCAAATATAG-3′
*Vegfa*	5′-ATGCGGATCAAACCTCACCAA-3′	5′-TTAACTCAAGCTGCCTCGCCT-3′

**Table 2:** Sequences of primers used for miRNA-specific qPCR analysis

miRNA name	Forward primer
miR-1	5′-GCTGGAATGTAAAGAAG TATGTAT-3′
miR-133a/b	5′-TGGTCCCCTTCAACCAGCTGT-3′
miR-206	5′-TGGAATGTAAGGAAGTGTGTGG-3′
U6	5′-CGCAAGGATGACACGCAAATTC-3′

### Analysis of mononucleated cell populations in skeletal muscles by flow cytometry

Samples for flow cytometry were prepared as described previously [9, 10]. Briefly, mice were euthanized and perfused immediately with saline containing 0.5 U/ml heparin through the left ventricle. Then, hind limbs muscles were excised, weighted, minced, and digested at 37 °C for 45 min in a solution containing collagenase IV (5 mg/ml; Gibco; Invitrogen) and dispase (1.2 U/ml; Gibco; Invitrogen). Digested muscles were passed through 100 μm cell strainer, washed with PBS, pelleted after centrifugation, and resuspended in PBS + 2% fetal bovine serum. Subsequently, the number of cells per milliliter was calculated using the Bürker chamber. Samples were stained with the following antibodies: rat anti-mouse CD45-APC-eFluor780 (30-F11, eBioscience), rat anti-mouse CD31-PE (MEC 13.3, BD Bioscience), rat anti-mouse CD34-Alexa Fluor 700 (RAM 34, eBioscience), rat anti-mouse Ly6A/E-PE-Cy7 (Sca-1; D7, eBioscience), rat anti-mouse α7-integrin-PE (334908, R&D Systems)—to analyze muscle SCs and fibro-adipogenic progenitors (FAPs). After fixation and permeabilization, mouse anti-MyoD (G-1, Santa Cruz Biotechnology) and rat anti-mouse Ki-67-FITC (SolA15, eBioscience) were applied. Subsequently, to detect MyoD, secondary goat anti-mouse Alexa Fluor 568 (Life Technologies) antibody was added. Rat anti-mouse CD45-APC-eFluor780 (30-F11, eBioscience), rat anti-mouse CD11b-Alexa Fluor 700 (M1/70, eBioscience), rat anti-mouse F4/80-APC (BM8, eBioscience), rat anti-mouse MHCII-PE-Cy7 (M5/114.15.2, BD Bioscience), rat anti-mouse CD206-PerCP/Cy5.5 (C068C2, BioLegend), rat anti-mouse Ly6C-AlexaFluor488 (HK 1.4, BD Biosciences), and rat anti-mouse Ly6G-PE (1A8, BioLegend) were used to analyze macrophages, whereas rat anti-mouse CD45-APC-eFluor780 (30-F11, eBioscience), hamster anti-mouse CD3e-PE-Cy7 (145-2C11, eBioscience), mouse anti-mouse NK1.1-FITC (PK 136, BioLegend), rat anti-mouse CD4-PerCP/Cy5.5 (RM 4-5, BD Biosciences), and rat anti-mouse CD8a-Alexa Fluor 700 (53-6.7, BioLegend)—to analyze lymphocyte populations and NK-cells. Before flow cytometry analysis, all cells were additionally stained with Hoechst 33342 (10 μg/ml). Data were acquired with a Fortessa flow cytometer (BD Biosciences) and analyzed using the FACSDiva software (BD Biosciences). Gates were set based on the appropriate fluorescent minus one (FMO) controls. In the case of MyoD staining, FMO controls were performed for each mouse used in the experiment. Results are presented as a cell number per milligram of tissue.

### Protein isolation

Total protein was isolated from snap-frozen GM by homogenization in lysis buffer—PBS containing inhibitors of proteinases (Roche) and 1% Triton X-100 (BioShop) using TissueLyser (Qiagen). Samples were then incubated on ice for 30 min, centrifuged (8000×*g*, 10 min, 4 °C), supernatants were collected and stored at −80 °C.

### Protein analysis

To assess monocyte chemoattractant protein 1 (MCP-1) and vascular endothelial growth factor A (VEGF) protein level, GM lysates were subjected to Luminex^TM^ measurement according to the manufacturer’s instructions (Life Technologies) whereas osteopontin concentration in plasma was determined by ELISA following the vendor’s protocol (R&D System). The results from MCP-1 and VEGF measurement were calculated as pg/mg of total protein and the level of osteopontin was shown in ng/ml.

### Statistics

Data are presented as mean ± SEM and analyzed with the unpaired two-tailed Student’s *t* test to determine differences between two groups or one-way ANOVA followed by Tukey’s post hoc test for multiple groups. *p* ≤ 0.05 was considered as significant. Grubb’s test was used to identify significant outliers, GraphPad Prism for graphs and statistical analyses.

## Results

### Lack of transcriptionally active Nrf2 enhances skeletal muscle damage after CTX-induced injury

**Fig. 1:**
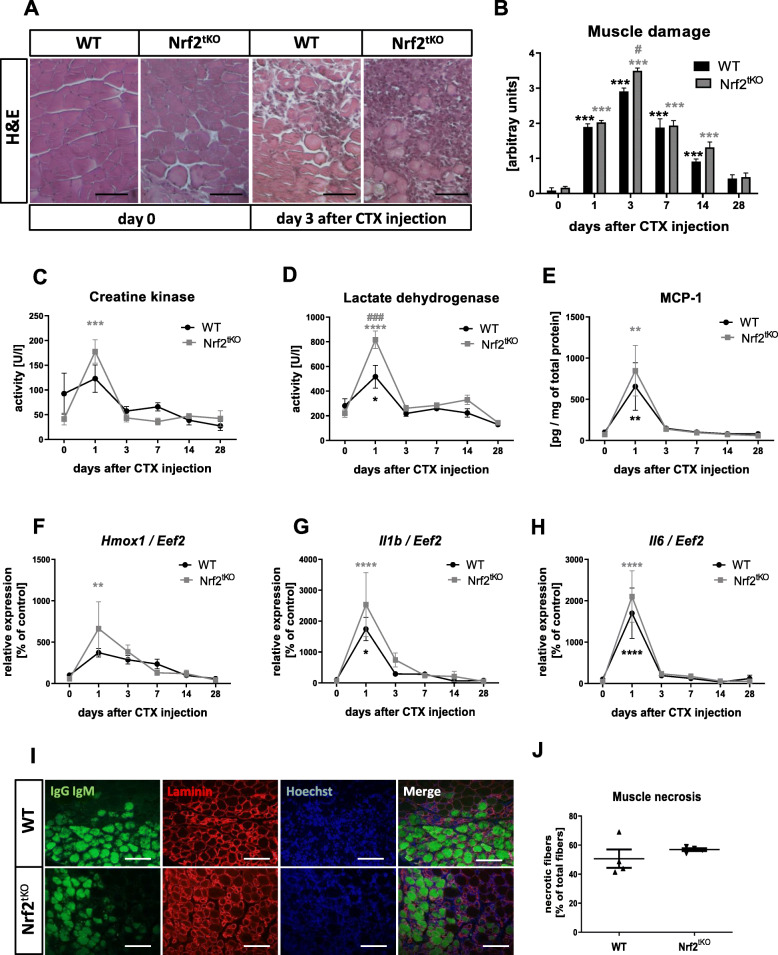
CTX-induced injury in GM of WT and Nrf2^tKO^ animals. (**a**) Representative photos and (**b**) semi-quantitative assessment of GM damage based on H&E staining; *n* = 4-6. The activity of (**c**) CK and (**d**) LDH in plasma; activity assay; *n* = 4-5. (**e**) MCP-1 protein level in GM, Luminex^TM^; *n* = 4-5. (**f**) *Hmox1*, (**g**) *Il1b*, (**h**) *Il6* level in GM; qRT-PCR; *n* = 4-6. (**i**) Representative photos of microscopic assessment of myofiber necrosis in GM and (**j**) quantification of the staining; *n* = 4. The data are presented as mean +/− SEM; *p ≤ 0.05; **p ≤ 0.01; ***p ≤ 0.001; ****p ≤ 0.0001 vs. day 0; #*p* ≤ 0.05; ###*p* ≤ 0.001 vs. WT, one-way ANOVA with Tukey’s post hoc test. The scale bars represent 100 μm

To analyze the effect of Nrf2 transcriptional deficiency during acute muscle damage, we examined inflammatory reaction and muscle degeneration as well as regeneration in the model of CTX-induced myoinjury. The level of muscle damage and inflammatory infiltration evaluated based on H&E staining (Fig. 1a, b) was significantly higher in Nrf2^tKO^ mice on the 3rd day after muscle damage. Although the activity of CK (Fig. 1c) was increased in Nrf2^tKO^ animals on day 1 after injection, a statistically significant difference between WT and Nrf2^tKO^ mice was not evident. On the other hand, the level of LDH (Fig. 1d) was significantly elevated in mice of both genotypes, and additionally, it was much higher in Nrf2^tKO^ animals in comparison to WT on the 1st day after CTX injection. Moreover, we have shown increased protein level of pro-inflammatory cytokine MCP-1 (Fig. 1e) and mRNA level of *Hmox1* (Fig. 1f), *Il1b* (Fig. 1g) and *Il6* (Fig. 1h) on the 1st day after CTX injection in both genotypes. Furthermore, IHF analysis of necrotic fibers on the 3rd day after myoinjury did not reveal differences between genotypes (Fig. 1i, j).

### Muscle regeneration is not affected in the absence of transcriptionally active Nrf2 following CTX-induced injury

**Fig. 2:**
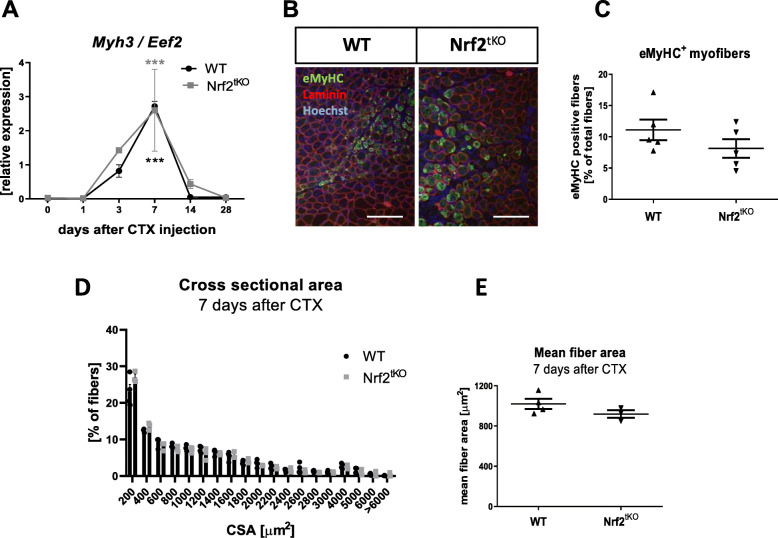
Muscle regeneration in GM of WT and Nrf2^tKO^ animals after CTX-induced injury. (**a**) *Myh3* level in GM; qRT-PCR; *n* = 4-6. (**b**) Representative photos of immunofluorescent staining for eMyHC and (**c**) quantification of the percentage of eMyHC positive myofibers; *n* = 5. (**d**) Cross sectional area of fibers 7 days after CTX; *n* = 3-4. (**e**) Mean fiber area 7 days after CTX; *n* = 3-4. The data are presented as mean +/− SEM; ***p ≤ 0.001 vs. day 0, one-way ANOVA with Tukey’s post hoc test. The scale bars represent 100 μm

To assess the role of Nrf2 during muscle regeneration following the acute muscle injury caused by CTX injection, we examined the mRNA level of *Myh3* and the number of eMyHC^+^ myofibers. Following muscle damage, we observed a higher level of *Myh3* in both WT and Nrf2^tKO^ animals on the 7th day after injury, however, there were no differences among genotypes in all analyzed timepoints (Fig. 2a). Accordingly, the number of eMyHC^+^ fibers (Fig. 2b, c), CSA (Fig. 2d), and mean fiber area (Fig. 2e) were similar on day 7 after injury.

### Transcriptional deficiency of Nrf2 does not aggravate dystrophic phenotype in *mdx* mice

**Fig. 3:**
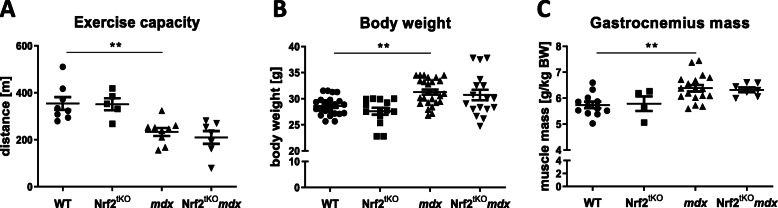
General phenotype of non-exercised WT, Nrf2^tKO^, *mdx*, and Nrf2^tKO^*mdx* mice. (**a**) Muscle performance; downhill running treadmill test; *n* = 5-9. (**b**) Body weight of mice; *n* = 14-26; (**c**) Gastrocnemius muscle mass; *n* = 4-18. The data are presented as mean +/− SEM; **p ≤ 0.01, one-way ANOVA with Tukey’s post hoc test

To investigate the role of Nrf2 in chronic muscle injury, we generated dystrophic mice lacking the transcriptional activity of Nrf2 (Nrf2^tKO^*mdx*). In order to determine whether the lack of Nrf2 can affect exercise performance, mice were subjected to a downhill treadmill run to exhaustion. As shown by us [9] and others [24] previously, and confirmed in the present study, *mdx* mice were able to run a shorter distance than WT (Fig. 3a). However, we did not observe an effect of the transcriptional deficiency of Nrf2 on the running pattern. The exercise capacity of Nrf2^tKO^ animals was comparable to WT mice and Nrf2^tKO^*mdx* mice run similar distance as *mdx* counterparts (Fig. 3a). Body weight (Fig. 3b) and GM mass (Fig. 3c) were significantly increased in *mdx* mice in comparison to healthy animals, while in Nrf2^tKO^*mdx*, no striking differences compared with age-matched *mdx* mice were found.

### Transcriptional knockout of Nrf2 does not exacerbate muscle degeneration in non-exercised *mdx* mice

**Fig. 4:**
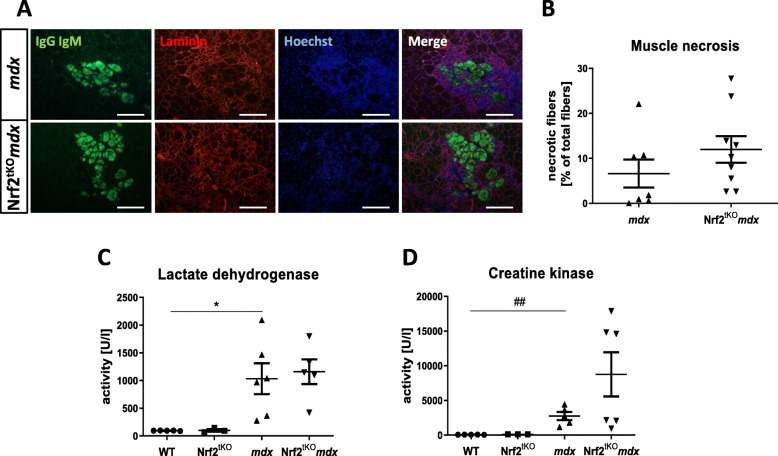
Muscle degeneration of non-exercised WT, Nrf2^tKO^, *mdx*, and Nrf2^tKO^*mdx* mice. (**a**) Microscopic assessment of myofiber necrosis in GM using immunofluorescent staining of IgM and IgG (green) binding and (**b**) its calculation; *n* = 7-9. The activity of (**c**) LDH and (**d**) CK in plasma; activity assay; *n* = 3-6. The data are presented as mean +/− SEM; *p ≤ 0.05, one-way ANOVA with Tukey’s post hoc test; ##*p* ≤ 0.01 Student’s *t* test. The scale bars represent 100 μm

Muscle degeneration was evaluated based on the percentage of necrotic fibers in GM as well as the plasma level of CK and LDH, typical markers of muscle damage. Neither the number of necrotic fibers (Fig. 4a, b) nor LDH (Fig. 4c) and CK (Fig. 4d) activity was changed between dystrophic mice additionally lacking Nrf2 and *mdx* animals, indicating a comparable level of muscle injury. As suspected, plasma LDH and CK levels of *mdx* mice were elevated compared with those of WT mice (Fig. 4c, d, respectively).

### Lack of Nrf2 transcriptional activity does not aggravate the inflammatory reaction in non-exercised dystrophic skeletal muscles

**Fig. 5:**
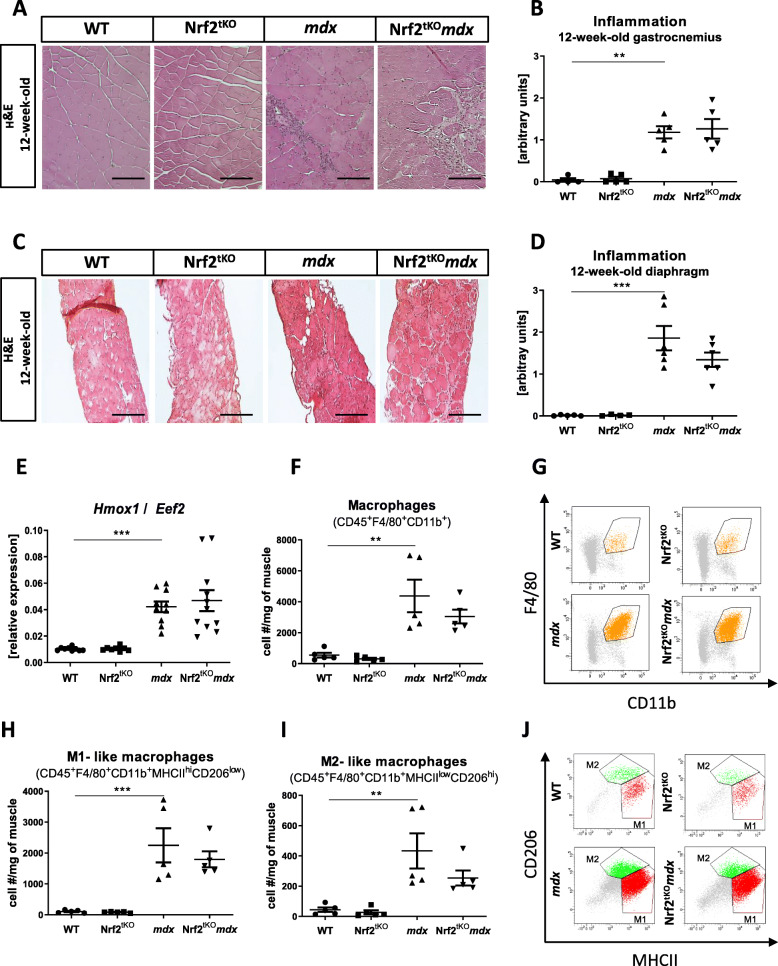
Infiltration of non-exercised WT, Nrf2^tKO^, *mdx*, and Nrf2^tKO^*mdx* hind limb muscle and diaphragm with leukocytes and macrophages. (**a**) Representative photos and (**b**) semi-quantitative analysis of inflammation in GM based on H&E staining; *n* = 4-6. (**c**) Representative photos and (**d**) semi-quantitative analysis of inflammation in diaphragm based on H&E staining; *n* = 4-6. (**e**) *Hmox1* level in GM; qRT-PCR; *n* = 8-11. (**f**) Number of macrophages (CD45^+^F4/80^+^CD11b^+^); flow cytometry; *n* = 5; (**g**) representative two-parameters flow cytometry dot plots. (**h**) Number of M1-like macrophages (CD45^+^F4/80^+^CD11b^+^MHCII^hi^CD206^lo^) and (**i**) M2-like macrophages (CD45^+^F4/80^+^CD11b^+^MHCII^lo^CD206^hi^); flow cytometry; *n* = 5; (**j**) representative two-parameters flow cytometry dot plots. The data are presented as mean +/− SEM; **p ≤ 0.01; ***p ≤ 0.001, one-way ANOVA with Tukey’s post hoc test. The scale bars represent 100 μm

Since Nrf2 has been reported as a master regulator of anti-oxidative responses that contributes to the anti-inflammatory process [30, 31], we have assessed whether it can affect the inflammatory reaction in skeletal muscle in our experimental conditions. Analysis of H&E staining demonstrated that *mdx* mice lacking transcriptional activity of Nrf2 had a similar inflammation score to *mdx* mice in both GM (Fig. 5a, b) and diaphragm (Fig. 5c, d). Moreover, the expression of *Hmox1*, an anti-inflammatory factor shown by us to be upregulated in dystrophic muscles [9], was again potently elevated in GM of *mdx* mice. However, it was the same in Nrf2^tKO^*mdx* mice, indicating that Nrf2 transcriptional activity is dispensable for *Hmox1* induction in the muscles (Fig. 5e).

To shed more light on the inflammatory status, we have performed a comprehensive analysis of different leukocyte populations within hind limb muscles using flow cytometry. *Mdx* mice demonstrated an abundance of macrophages defined as CD45^+^F4/80^+^CD11b^+^ cells. However, no further changes in the infiltration of this population into skeletal muscle were caused by Nrf2 transcriptional deficiency (Fig. 5 f, g). Due to the diverse functions of different subpopulations of macrophages [4], in the next step, we have investigated M1-like and M2-like macrophages, based on the gating strategy discriminating between MHCII and CD206 expression by CD45^+^F4/80^+^CD11b^+^ cells. The subsets of both M1-like (CD45^+^F4/80^+^CD11b^+^MHCII^hi^CD206^low^) and M2-like (CD45^+^F4/80^+^CD11b^+^MHCII^low^ CD206^hi^) macrophages were much more elevated in dystrophic mice in comparison to WT but the lack of transcriptionally active Nrf2 did not further change their number (Fig. 5h, i, j).

**Fig. 6:**
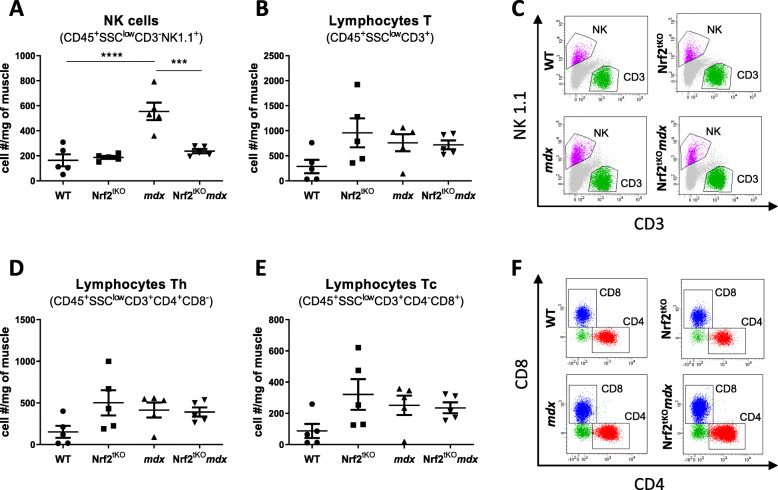
Infiltration of non-exercised WT, Nrf2^tKO^, *mdx*, and Nrf2^tKO^*mdx* hind limb muscle with NK cells and lymphocytes. (**a**) Number of NK cells (CD45^+^SSC^low^CD3^−^NK1.1^+^) and (**b**) lymphocytes T (CD45^+^SSC^low^CD3^+^); flow cytometry; (**c**) representative two-parameters flow cytometry dot plots. (**d**) Number of lymphocytes T_h_ (CD45^+^SSC^low^CD3^+^CD4^+^CD8^−^) and (**e**) T_c_ (CD45^+^SSC^low^CD3^+^CD4^−^CD8^+^); flow cytometry; (**f**) representative two-parameters flow cytometry dot plots. The data are presented as mean +/− SEM; *n* = 5; ***p ≤ 0.001; ****p ≤ 0.0001, one-way ANOVA with Tukey’s post test

The number of NK cells (CD45^+^SSC^low^CD3^−^NK1.1^+^) was significantly higher in *mdx* mice in comparison to WT. Conversely, dystrophic mice additionally lacking Nrf2 transcriptional activity exhibited decreased NK number reaching a similar number to the one observed in WT animals (Fig. 6a, c). The number of lymphocytes T (CD45^+^SSC^low^CD3^+^) (Fig. 6b, c), T helper (T_h_; CD45^+^SSC^low^CD3^+^CD4^+^CD8^−^) (Fig. 6d, f), and T cytotoxic (T_c_; CD45^+^SSC^low^CD3^+^CD4^−^CD8^+^) (Fig. 6e, f) were the same among all four genotypes.

### The role of Nrf2 transcriptional deficiency on muscle fibrosis in non-exercised *mdx* mice

**Fig. 7:**
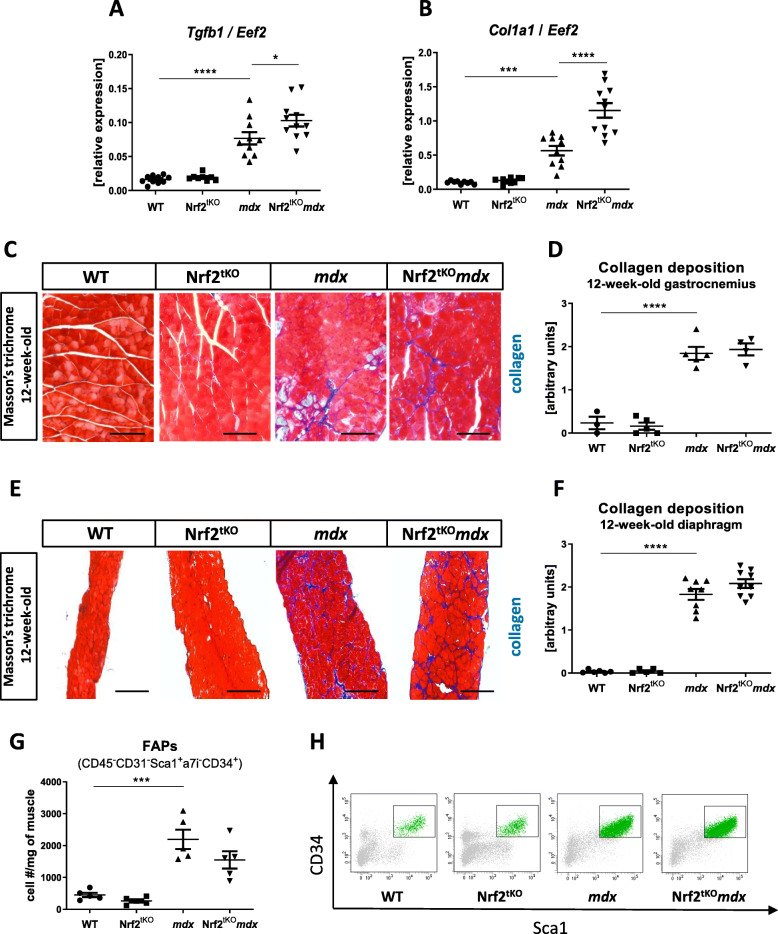
Fibrosis in non-exercised WT, Nrf2^tKO^, *mdx*, and Nrf2^tKO^*mdx* hind limb muscles and diaphragm. (**a**) *Tgfb1*, (**b**) *Col1a1* levels in GM; qRT-PCR; *n* = 9-11. (**c**) Representative photos and (**d**) semi-quantitative analysis of collagen deposition in GM based on Masson’s trichome staining; *n* = 3-5. (**e**) Representative photos and (**f**) semi-quantitative analysis of collagen deposition in diaphragm based on Masson’s trichome staining; *n* = 5-8. (**g**) Number of FAPs (CD45^+^CD31^−^Sca1^+^a7i^−^CD34^+^); flow cytometry; *n* = 5; (**h**) representative two-parameters flow cytometry dot plots. The data are presented as mean +/− SEM; *p ≤ 0.05; ***p ≤ 0.001; ****p ≤ 0.0001, one-way ANOVA with Tukey’s post hoc test. The scale bars represent 100 μm

We have found that transforming growth factor beta-1 (*Tgfb1*) and collagen type I alpha 1 (*Col1a1*) were upregulated in *mdx* vs. WT animals and were further elevated in *mdx* mice lacking additionally transcriptionally active Nrf2 (Fig. 7a, b), suggesting that the transcriptional deficiency of Nrf2 could enhance fibrosis. To confirm those results, a histological analysis of collagen deposition based on Masson’s trichrome staining was performed. Accordingly, endomysial collagen content was significantly elevated in both GM (Fig. 7c, d) and diaphragm (Fig. 7e, f) of dystrophic mice; however, it was not further exacerbated in *mdx* mice lacking transcriptionally active Nrf2. Moreover, as FAPs are also involved in the progression of DMD [32], we checked their numbers using flow cytometry. Our results showed that FAPs, defined as CD45^−^CD31^−^Sca1^+^a7i^−^CD34^+^ cells, were upregulated by dystrophin deficiency, but their numbers were not further affected by the lack of transcriptionally active Nrf2 (Fig. 7g, h).

### A decrease in the expression of angiogenic mediators in non-exercised *mdx* mice is not affected by the lack of transcriptionally active Nrf2

**Fig. 8:**
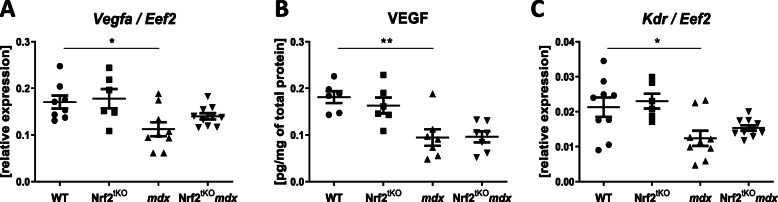
Expression of angiogenic mediators in skeletal muscle of non-exercised WT, Nrf2^tKO^, *mdx*, and Nrf2^tKO^*mdx* mice. The expression of (**a**) *Vegfa* in GM; qRT-PCR; *n* = 6-10 and (**b**) VEGF protein level in GM, Luminex^TM^; *n* = 6-7. (**c**) The expression of VEGF receptor (*Kdr*); qRT-PCR; *n* = 6-10. The data are presented as mean ± SEM. *p ≤ 0.05; **p ≤ 0.01, one-way ANOVA with Tukey’s post hoc test

Dysregulation of angiogenesis may greatly contribute to DMD progression [33]. Moreover, Nrf2 was shown to regulate neovascularization and to exert a pivotal role in angiogenic signal transduction and angiogenic potential of not only endothelial cells itself but also bone marrow-derived proangiogenic cells [34]. Therefore, we aimed to investigate the angiogenic signaling in our model. Firstly, we have checked the mRNA and protein level of the major proangiogenic factor, VEGF, in GM of mice of all genotypes. The mRNA expression was diminished in *mdx* mice but no further changes were observed in double knockouts (Fig. 8a). Concomitantly, the level of VEGF protein was potently downregulated in dystrophic GM; however, the lack of transcriptionally active Nrf2 did not affect this production in both healthy and dystrophic mice (Fig. 8b). A similar trend of changes was found when the expression of *Kdr*, a receptor for VEGF was evaluated (Fig. 8c).

### Nrf2 transcriptional deficiency does not affect the number and proliferation of muscle SCs but it may influence muscle regeneration in non-exercised dystrophic animals

**Fig. 9:**
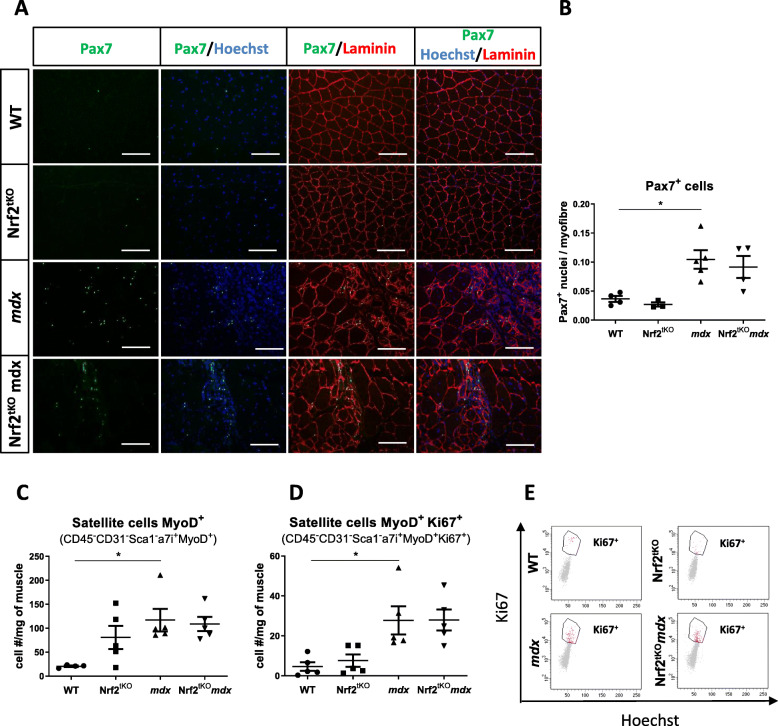
Number and proliferation of SCs from non-exercised WT, Nrf2^tKO^, *mdx*, and Nrf2^tKO^*mdx* hind limb muscles. (**a**) Pax7 staining in GM; representative photos. (**b**) Quantification of the ratio of Pax7^+^ cells per myofibre; *n* = 3-5. (**c**) Number of MyoD^+^ SCs (CD45^−^CD31^−^Sca1^−^α7integrin^+^MyoD^+^); flow cytometry; *n* = 4-5. (**d**) Number of proliferating MyoD^+^ SCs (CD45^−^CD31^−^Sca1^−^α7integrin^+^MyoD^+^Ki67^+^); flow cytometry; *n* = 5; (**e**) representative two-parameters flow cytometry dot plots. The data are presented as mean +/− SEM; *p ≤ 0.05; one-way ANOVA with Tukey’s post hoc test. The scale bars represent 100 μm

The number of SCs evaluated based on IHF staining and calculation of the ratio of Pax7-positive nuclei to myofibers revealed an increased number of Pax7^+^ cells in dystrophin-deficient mice in comparison to healthy ones, however, the additional effect of the lack of transcriptionally active Nrf2 was not observed (Fig. 9a, b). Furthermore, flow cytometry analysis demonstrated a considerable increase in the number of MyoD-positive SCs (CD45^-^CD31^−^Sca1^−^α7integrin^+^MyoD^+^) in *mdx* mice, but it was not further changed by Nrf2 transcriptional deficiency (Fig. 9c). We have also checked the proliferation of MyoD-positive SCs by cytofluorimetric analysis of cells expressing Ki67. Significantly enhanced proliferation of MyoD^+^ SCs in dystrophic mice compared to healthy animals was observed. This was not further potentiated in Nrf2^tKO^*mdx* mice (Fig. 9d, e).

**Fig. 10:**
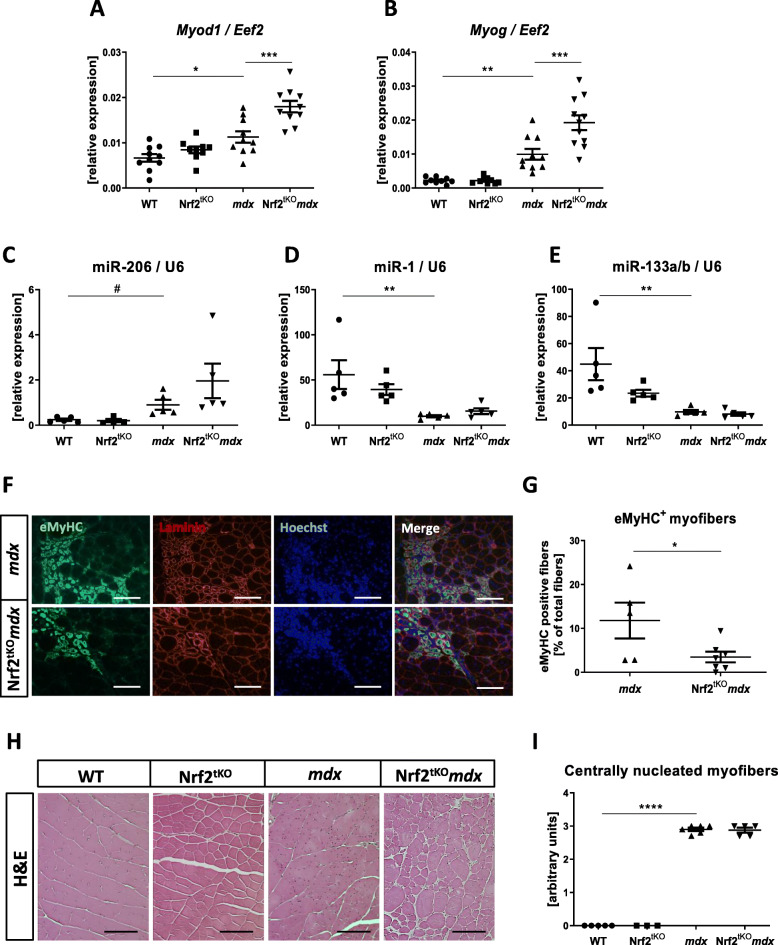
Regeneration of GM of non-exercised WT, Nrf2^tKO^, *mdx*, and Nrf2^tKO^*mdx* mice. (**a**) *Myod1*, (**b**) *Myog*, (**c**) miR-206, (**d**) miR-1, (**e**) miR-133a/b level in GM; qRT-PCR; *n* = 9-11. (**f**) Representative photos of immunofluorescent staining for eMyHC and (**g**) quantification of the percentage of eMyHC positive myofibers; *n* = 5-7. (**h**) Representative photos and (**i**) semi-quantitative analysis of centrally nucleated myofibers in GM based on H&E staining; *n* = 3-6. The data are presented as mean +/− SEM; *p ≤ 0.05; **p ≤ 0.01; ***p ≤ 0.001; ****p ≤ 0.0001, one-way ANOVA with Tukey’s post hoc test; #*p* ≤ 0.05 Student’s *t* test. The scale bars represent 100 μm

Although there was no effect of the lack of transcriptionally active Nrf2 on the number and proliferation of SCs, we have shown that the regeneration process is affected in the course of chronic injury in dystrophic mice, and what is more—it is additionally altered by the Nrf2 status. Accordingly, dystrophic mice showed higher expression of myogenic regulatory factors such as myogenic differentiation 1 (*Myod1*) and myogenin (*Myog*) than their healthy counterparts, and the expression of those factors was further enhanced by Nrf2 transcriptional deficiency (Fig. 10a, b).

Additionally, we have checked the mRNA level of muscle-specific microRNAs which also contribute to the process of muscle regeneration [35]. Expression of miR-206 (Fig. 10c) was elevated in *mdx* mice in comparison to age-matched WT animals whereas miR-1 (Fig. 10d) and miR-133a/b (Fig. 10e) showed the opposite pattern. However, none of them were affected by Nrf2 transcriptional deficiency.

Finally, the number of myofibers expressing eMyHC, the marker of regeneration, was diminished in *mdx* mice additionally lacking transcriptionally active Nrf2 in comparison to *mdx* counterparts (Fig. 10f, g). However, histological analysis of centrally nucleated fibers did not show differences between *mdx* and Nrf2^tKO^*mdx* animals (Fig. 10h, i).

### Chronic treadmill exercise aggravates skeletal muscle degeneration and inflammation in dystrophic animals lacking transcriptional activity of Nrf2

**Fig. 11:**
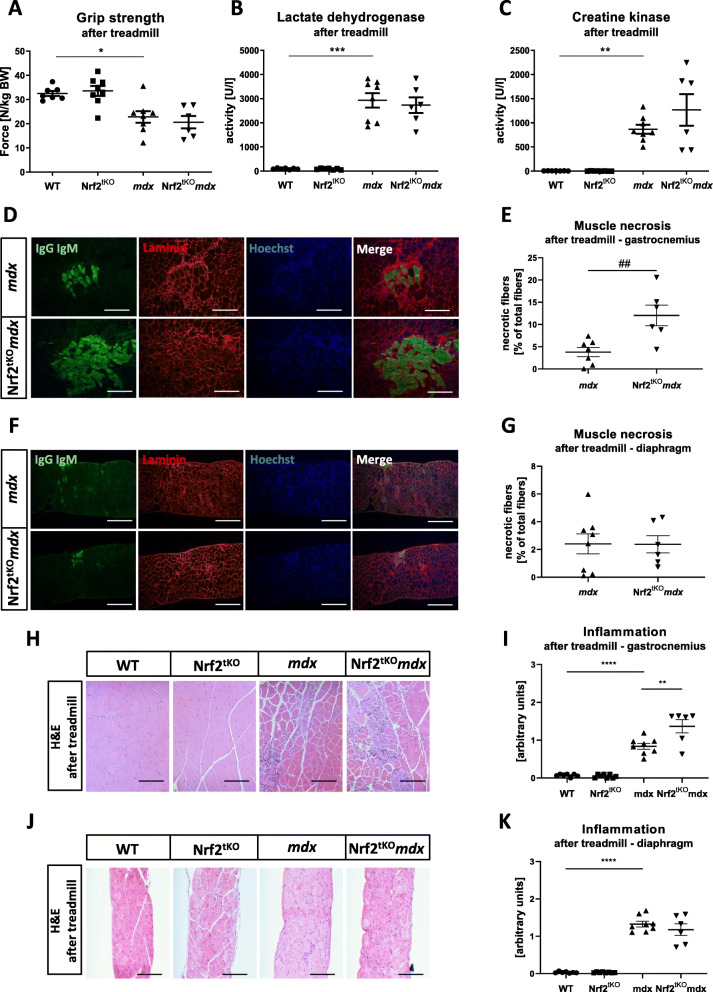
Muscle degeneration and inflammation of WT, Nrf2^tKO^, *mdx*, and Nrf2^tKO^*mdx* mice after the long-term treadmill. (**a**) Grip strength test; *n* = 6-8. The activity of (**b**) LDH and (**c**) CK in plasma; activity assay; *n* = 6-8. (**d**) Microscopic assessment of myofiber necrosis in GM using immunofluorescent staining of IgM and IgG (green) binding and (**e**) its calculation; *n* = 6-8. (**f**) Microscopic assessment of myofiber necrosis in diaphragm using immunofluorescent staining of IgM and IgG (green) binding and (**g**) its calculation; *n* = 6-8. (**h**) Representative photos and (**i**) semi-quantitative analysis of inflammation in GM based on H&E staining; *n* = 6-8. (**j**) Representative photos and (**k**) semi-quantitative analysis of inflammation in diaphragm based on H&E staining; *n* = 6-8. The data are presented as mean +/− SEM; *p ≤ 0.05, **p ≤ 0.01; ***p ≤ 0.001, ****p ≤ 0.0001, one-way ANOVA with Tukey’s post hoc test; ##*p* ≤ 0.01 Student’s *t* test. The scale bars represent 100 μm

In order to identify the role of Nrf2 under aggravated dystrophic conditions, we have analyzed muscle functionality, degeneration, and inflammation after 4 weeks of chronic treadmill exercises. Firstly, reduced muscle function measured by the grip strength test in *mdx* mice in comparison to healthy counterparts was shown; however, it was not additionally altered by the transcriptional deficiency of Nrf2 (Fig. 11a). Moreover, the activity of LDH and CK in plasma did not differ in Nrf2^tKO^*mdx* vs. *mdx* animals (Fig. 11b, c, respectively).

The number of necrotic fibers (Fig. 11d, e) was significantly higher in GM of *mdx* mice in comparison to WT, and interestingly, it was further increased in Nrf2^tKO^*mdx* animals in comparison to *mdx* (Fig. 11d, e). However, this was not observed in the diaphragm (Fig. 11f, g). Furthermore, the same pattern was noticed in the case of inflammatory infiltration evaluated through H&E staining—higher inflammatory extent in Nrf2^tKO^*mdx* animals in comparison to *mdx* in GM (Fig. 11h, i), but not in the diaphragm (Fig. 11j, k).

**Fig. 12:**
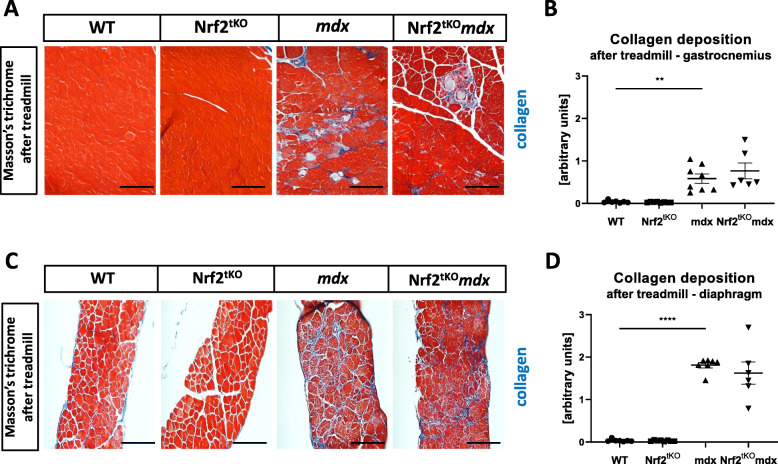
Fibrosis in WT, Nrf2^tKO^, *mdx*, and Nrf2^tKO^*mdx* hind limb muscle and diaphragm after the long-term treadmill. (**a**) Representative photos and (**b**) semi-quantitative analysis of collagen deposition in GM based on Masson’s trichome staining; *n* = 6-8. (**c**) Representative photos and (**d**) semi-quantitative analysis of collagen deposition in diaphragm based on Masson’s trichome staining; *n* = 6-8. The data are presented as mean +/− SEM **p ≤ 0.01; ****p ≤ 0.0001, one-way ANOVA with Tukey’s post hoc test; the scale bars represent 100 μm

As muscle fibrosis might be enhanced by chronic treadmill exercises [36], we have checked if it is altered by an additional lack of Nrf2. Collagen deposition analysis based on Masson’s trichrome staining revealed increased collagen content in *mdx* mice; however, it was not further altered by the transcriptional deficiency of Nrf2, neither in GM (Fig. 12a, b) nor the diaphragm (Fig. 12c, d). Moreover, the plasma level of osteopontin, a biomarker of DMD associated with fibrosis [37], was comparable between *mdx* and Nrf2^tKO^*mdx* mice (Fig. S1 A).

### Inflammation and fibrosis are not affected in 24-week-old dystrophic mice by the lack of transcriptional activity of Nrf2

**Fig. 13:**
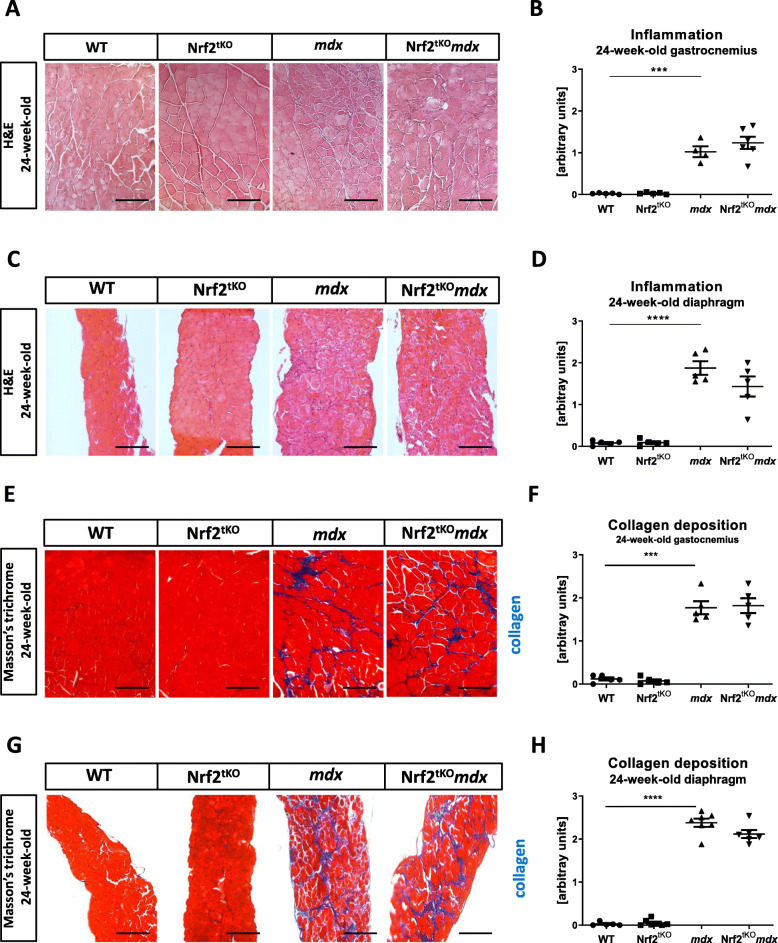
Inflammation and fibrosis in muscles of non-exercised 24-week-old WT, Nrf2^tKO^, *mdx*, and Nrf2^tKO^*mdx* mice. (**a**) Representative photos and (**b**) semi-quantitative analysis of inflammation in GM based on H&E staining; *n* = 4-6. (**c**) Representative photos and (**d**) semi-quantitative analysis of inflammation in diaphragm based on H&E staining; *n* = 5. (**e**) Representative photos and (**f**) semi-quantitative analysis of collagen deposition in GM based on Masson’s trichome staining; *n* = 5. (**g**) Representative photos and (**h**) semi-quantitative analysis of inflammation in diaphragm based on Masson’s trichome staining; *n* = 5-7. The data are presented as mean +/− SEM; ***p ≤ 0.001; ****p ≤ 0.0001, one-way ANOVA with Tukey’s post hoc test; the scale bars represent 100 μm

To assess the role of Nrf2 transcriptional deficiency on inflammation and fibrosis processes in old mice, we have performed H&E and Masson’s trichrome staining on GM and diaphragm of 24-week-old animals. We have observed increased inflammatory infiltration based on H&E staining in both gastrocnemius (Fig. 13a, b) and diaphragm (Fig. 13c, d) muscles in *mdx* mice in comparison to their healthy counterparts; however, it was not further changed by the lack of transcriptionally active Nrf2. Similarly, the same trend was observed in the case of fibrosis content, and analyzed based on Masson’s trichrome staining. Increased collagen deposition in GM (Fig. 13e, f) and diaphragm (Fig. 13g, h) of dystrophic mice was demonstrated, but it was not altered by the lack of Nrf2 transcriptional activity in both muscles.

## Discussion

DMD is still an incurable disease with very limited treatment possibilities, including corticosteroids [38]. However, despite having a beneficial effect on muscle function, these drugs cause many side effects [39]. Therefore, identifying new targets for anti-inflammatory treatment may contribute to the development of novel therapeutic strategies. Taking into consideration the pleiotropic activity of Nrf2, which drives the expression of anti-inflammatory, anti-oxidant, and cytoprotective genes, we aimed to investigate its role in the acute muscle injury induced by CTX injection and in the progression of DMD using two different mouse models—*mdx* and exacerbated dystrophic phenotype—*mdx* mice subjected to the long-term treadmill exercise. In our study, we found that the absence of transcriptionally active Nrf2 may be associated with increased muscle damage after acute muscle injury following CTX injection and under aggravated dystrophic conditions, however, it did not significantly affect the pathophysiological hallmarks of DMD progression.

Due to the disruption of the DGC complex and increased sarcolemmal permeability in DMD [40], serum proteins such as IgG and IgM, which are typically found only in circulation, are accumulated in damaged, necrotic myofibers [41]. We have shown that the number of such necrotic fibers does not differ between non-exercised *mdx* and Nrf2^tKO^*mdx* mice. Although we have demonstrated higher levels of CK and LDH in the serum of non-exercised *mdx* mice in comparison to healthy counterparts, their levels were not further elevated in dystrophic mice lacking transcriptionally active Nrf2. We have also performed such analysis after a single CTX injury, a model where the kinetics of degeneration and inflammation processes are more stable than during DMD development. Although a similar experimental model was previously utilized by Shelar et al. [42], the degeneration has not been checked. In our hand, Nrf2 transcriptional deficiency was associated with the aggravation of LDH activity in plasma on 1st day after injury and enhanced muscle damage on 3rd day following CTX injury. Hence, it might be hypothesized that at the stage of massive macrophages infiltration after CTX injury occurring just on day 3 [43], the lack of Nrf2 manifests more profoundly, impairing the proper inflammatory response. However, this hypothesis would require further verification.

Inflammation is one of the most prominent features of dystrophic muscles. In our studies, contrary to the expectations, inflammation was not exacerbated in dystrophic muscles lacking transcriptionally active Nrf2 both when total inflammatory extent was evaluated on histological sections and by detailed analysis of immune subtypes by flow cytometry. Of note, despite the fact T_h_ and T_c_ lymphocytes contribute to the pathogenesis of DMD [6] and their functions may be regulated by Nrf2 [44], their numbers were changed neither by dystrophin nor Nrf2 deficiency in non-exercised animals. An interesting finding of our study is related to the NK cells’ abundance—their number was significantly decreased in Nrf2^tKO^*mdx* mice in comparison to *mdx*, in which they are elevated in the muscles [45]. Hence, our observations are consistent with previous reports showing an increased percentage of NK cells following activation of Nrf2 [46].

Petrillo et al. demonstrated a higher expression of Nrf2 and its target, HO-1, in muscle biopsies from DMD patients in comparison to healthy individuals [15]. In our model, we observed an elevated level of HO-1 in the CTX-injured muscles on day 1 after injection, as well as in the dystrophic, non-exercised mice, consistent with our previous results [9, 10]. However, the effect of Nrf2 deficiency on the level of this important inflammatory mediator has been noticed neither in Nrf2^tKO^ following CTX injury nor in non-exercised Nrf2^tKO^*mdx* indicating that HO-1 may be regulated independently of Nrf2. Indeed, such an Nrf2-independent regulation of HO-1, relying on, e.g., Foxo1 transcription factor, has been demonstrated in the muscles and SCs [9, 47].

Concomitantly, we have revealed that Nrf2 plays a dispensable role in endomysial fibrosis in dystrophic muscles, the process of accumulation of connective tissue, which is the characteristic attribute of DMD pathology [4], is associated with poor outcome [48] and linked to the increased expression of TGF-β1 and collagen [49–51]. We have observed elevated mRNA level of *Tgfb1* and *Col1a1* not only in non-exercised *mdx* animals but even at a higher degree in dystrophic mice additionally lacking transcriptionally active Nrf2, what is consistent with studies showing that Nrf2 acts as a protective agent against fibrosis [52–54]. However, although the lack of transcriptional activity of Nrf2 in dystrophic mice affected mRNA of fibrotic genes, it was not reflected in the collagen deposition in muscle’s histological sections. Moreover, the number of FAPs remained comparable between those two groups of animals. The observed differences may be caused by the fact that the mRNA level points to a particular time-point, whereas collagen deposition showed changes arising during disease progression.

Additionally, to fully understand the potential role of Nrf2 in DMD progression, we have studied the inflammation and fibrosis in older—24-week-old animals. Accordingly, differences between non-exercised *mdx* and Nrf2^tKO^*mdx* were observed neither in GM nor in the diaphragm. Importantly, our data are in line with the work by Takemoto et al. [55] what might indicate that older *mdx* mice are not a good model to study DMD progression due to disease stabilization at later stages [56–58]. Therefore, we have also looked at the role of Nrf2 in exacerbated dystrophic phenotype—mice subjected to long-term treadmill exercises. Interestingly, we have observed an increased number of necrotic fibers in GM of dystrophic mice in comparison to the diaphragm what is in line with the previous findings [59]. However, again, we did not find any changes in dystrophic muscle functionality and other pathological aspects of DMD driven by the lack of Nrf2 transcriptional activity except for the higher level of degeneration and inflammation in GM of Nrf2^tKO^*mdx* mice, but not in the diaphragm. The latter could be explained by the applied experimental protocol of chronic exercises with relatively low speed and maintaining horizontal orientation, which possibly did not influence the diaphragm, contrary to the application of more acute protocols [60].

Effective muscle regeneration is achieved by SCs [61]. In DMD, repeated cycles of muscle damage and regeneration disturbed the balance between self-renewal and differentiation, leading to premature depletion of the SCs pool [62]. We have shown that the absolute number of Pax7-positive SCs, as well as MyoD-positive SCs, was notably increased in dystrophic animals as in the previous studies performed on *mdx* mice [63]. Nevertheless, regardless of the method used, the number, as well as the proliferation of dystrophic SCs, was not additionally altered by Nrf2 transcriptional deficiency. It is well established that muscle regeneration is controlled by myogenic regulatory factors (MRFs) such as MyoD and myogenin. Their elevated level was observed by us in *mdx* mice and was further upregulated by additional Nrf2 transcriptional deficiency, the mechanism of which is currently investigated. Newly formed myofibers are characterized by the expression of unique myosin isoforms such as eMyHC. The number of eMyHC-positive myofibers was lower in *mdx* mice additionally lacking transcriptional activity of Nrf2 in comparison to *mdx*. However, because there are many repeated cycles of muscle damage and regeneration in DMD, it is difficult to conclude how Nrf2 affects this process based on transiently expressed eMyHC [64]. Therefore, we also checked muscle regeneration after acute muscle injury induced by CTX injection, a method that allows studying muscle regeneration in more controlled and reproducible conditions [65]. In the model of CTX-induced muscle injury, no effect of the lack of Nrf2 transcriptional deficiency was observed. This finding is consistent with previous studies where a similar level of regeneration in transcriptionally deficient Nrf2 and control mice was demonstrated [55]. Contrary, Shelar et al. presented delayed regeneration of tibialis anterior muscle in Nrf2-deficient mice [42], however, a few experimental differences (e.g., the age of animals and divergent muscles injected with various doses and types of CTX) may potentially explain discrepancies in the results obtained in both studies.

Overall, our results did not show the prominent effects of Nrf2 deficiency both in acute and in chronic muscle injury. However, it is still possible that beneficial effects could be observed through Nrf2 induction or overexpression. Sun et al. have shown that dystrophic phenotype may be markedly alleviated in *mdx* mice by treatment with sulforaphane (SFN) [16, 18], an isothiocyanate that activates Nrf2 by modifying Keap1 cysteines [66]. Moreover, several studies have outlined that some features of DMD might be improved by other anti-oxidant compounds like resveratrol [67] or curcumin [68], but their effect was not as strong as SFN [16, 18]. However, it is important to remember that such compounds might work not solely through activation of Nrf2, but they may also involve other mechanisms [69–71], and in the above studies the involvement of the Nrf2 pathway was not investigated in details.

## Conclusion

Lack of transcriptionally active Nrf2 is associated with slightly increased muscle damage after acute muscle injury caused by CTX injection as well as degeneration and inflammatory infiltration of the gastrocnemius muscle in dystrophic mice upon chronic treadmill exercises. Nevertheless, Nrf2 ablation does not significantly aggravate the most deleterious pathological events such as degeneration, inflammation, angiogenesis, and fibrotic scar formation as well as the number and proliferation of SCs in the non-exercised *mdx* mice model. Therefore, we conclude that the deficiency of Nrf2 transcriptional activity has no profound impact on muscle pathology in various models of muscle injury.

## Supplementary Information

**Additional file 1: Supplementary Figure 1. (A)** Osteopontin concentration in plasma of WT, Nrf2^tKO^, *mdx* and Nrf2^tKO^*mdx* after the long-term treadmill. ELISA; *n*=6-8. The data are presented as mean +/- SEM ***p≤0.001, one-way ANOVA with Tukey’s post-hoc test.

## Data Availability

The datasets used and/or analyzed during the current study are available from the corresponding author on reasonable request.

## Abbreviations

ARE: Anti-oxidant response element
BSA: Bovine serum albumin
CK: Creatine kinase
*Col1a1*: Collagen type I alpha 1
CSA: Cross-sectional area
CTX: Cardiotoxin
DGC: Dystrophin-glycoprotein complex
DMD: Duchenne muscular dystrophy
*Eef2*: Eukaryotic elongation factor 2
eMyHC: Embryonic myosin heavy chain
FMO: Fluorescent minus one
GM: Gastrocnemius muscle
H&E: Hematoxylin and eosin
*Hmox1*: Gene encoding HO-1
HO-1: Heme oxygenase-1
IACUC: Institutional Animal Care and Use Committee
IHF: Immunohistofluorescent
*Il1b*: Interleukin 1 beta
*Il6*: Interleukin 6
Keap1: Kelch-like ECH-associating protein 1
*Kdr*: Receptor for VEGF (VEGF-R2)
LDH: Lactate dehydrogenase
MCP-1: Monocyte chemoattractant protein 1
MRFs: Muscle regulatory factors
M.O.M: Mouse on mouse
*Myh3*: Myosin heavy chain 3
*Myog*: Myogenin
*Nfe2l2*: Gene encoding Nrf2
NK: Natural killer
Nrf2: Nuclear factor erythroid 2-related factor 2
OCT: Optimal cutting temperature compound
Pax7: Paired box 7
PBS: Phosphate-buffered saline
PCR: Polymerase chain reaction
qPCR: Quantitative PCR
ROS: Reactive oxygen species
SCs: Satellite cells
SEM: Standard error of the mean
T_c_: Lymphocytes T cytotoxic
TGF-β: Transforming growth factor-β
T_h_: Lymphocytes T helper
tKO: Transcriptional knockout
T_reg_: Lymphocytes T regulatory
VEGF: Vascular endothelial growth factor
WT: Wild type
