# Supplementary figures and images for: The role of Nrf2 in acute and chronic muscle injury
Source: Skelet Muscle. 2020 Dec 8;10:35. doi: 10.1186/s13395-020-00255-0 (PMC7722332; doi:10.1186/s13395-020-00255-0)

A

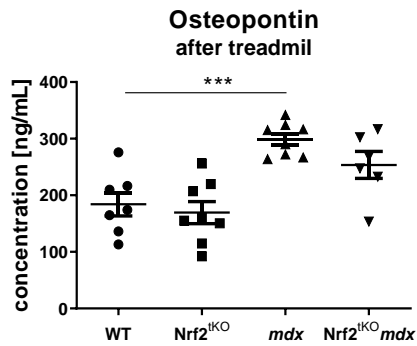

Supplement: Supplementary file 1 — Additional file 1: Supplementary Figure 1. (A) Osteopontin concentration in plasma of WT, Nrf2tKO, mdx and Nrf2tKOmdx after the long-term treadmill. ELISA; n=6-8. The data are presented as mean +/- SEM ***p≤0.001, one-way ANOVA with Tukey’s post-hoc test. [file 13395_2020_255_MOESM1_ESM.pdf]
